# Investigation of Occupational Health and Safety Levels in Genetic Disease Centers in Istanbul

**DOI:** 10.1002/jcla.70015

**Published:** 2025-03-26

**Authors:** Vedat Caner, Ferdi Tanir

**Affiliations:** ^1^ Istanbul Beykent University, Vocational School, Property Protection and Security Department Occupational Health and Safety Program Istanbul Turkey; ^2^ School of Medicine, Department of Internal Medicine, Department of Public Health Cukurova University Adana Turkey

**Keywords:** genetic disease diagnostic centers, laboratory hazards, occupational health and safety level

## Abstract

**Background:**

Genetic disorders significantly impact public health and quality of life, necessitating precise and timely diagnosis for effective risk management and treatment. Genetic diagnostic centers (GDCs) play a critical role in this process but face numerous occupational health and safety (OHS) challenges. The classification of GDCs based solely on biosafety levels is insufficient for assessing their overall OHS conditions. This study aims to systematically evaluate OHS practices in GDCs and propose a new classification approach based on hazard dimensions.

**Methods:**

This cross‐sectional study was conducted in 15 GDCs in Istanbul, including two public and 13 private facilities with 75 employees. Data were collected through a structured survey with 49 statements covering seven hazard dimensions. Regression and correlation analyses were used to assess the impacts and interrelationships of these dimensions on risk management. Principal Component Analysis (PCA) was applied for dimensionality reduction, and the k‐Nearest Neighbours (k‐NN) algorithm classified laboratories into safety levels.

**Results:**

Personal protective equipment had the highest impact on risk management (56.3%), while physical security had the lowest (34.8%). Among the 21 identified hazard relationships, 18 were very strong and three were strong. PCA reduced the data into three primary components, explaining 81.9% of the variance. The k‐NN algorithm achieved a classification accuracy of 93.33%, consolidating six hazard dimensions into three and categorizing centers into three safety levels.

**Conclusion:**

The findings emphasize the need for an updated OHS classification for GDCs beyond biosafety levels. Integrating hazard dimensions into safety assessments can improve risk management and enhance laboratory safety standards.

## Graphical abstract

Genetic diagnostic centers (GDCs) play a crucial role in hereditary disease management but face significant occupational health and safety (OHS) challenges. This study systematically evaluates OHS practices in 15 GDCs in Istanbul, using statistical analyses and machine learning techniques to classify laboratories based on hazard dimensions. The findings highlight the need for an updated classification system beyond biosafety levels to enhance laboratory safety and risk management.

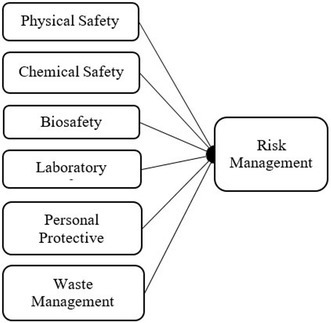

## 1 Introduction

Diagnostic studies for genetic disorders bring many benefits to people's lives. People with a family history of an inherited disease can access information about their own health and that of their children through genetic testing. The results of these tests allow for preventive treatment of diseases that have not yet developed. The ability to determine the likelihood of inherited disease also offers individuals the opportunity to modify their quality of life [1].

There are many hazards and risk factors involved in studies conducted in genetic disease diagnostic centers (GDDCs) [2, 3, 4]. To prevent hazards from becoming risks, risk assessments need to be performed. Although different risk assessment strategies exist, the general approach focuses on hazard identification and risk assessment [5]. If risks are not properly managed, near misses and workplace accidents can occur [6].

A study conducted at a university hospital in Istanbul found a significant positive correlation between age and occupational accidents. The average number of occupational accidents experienced by nurses was lower than that of doctors. In addition, the number of near misses reported by doctors was higher than that of nurses [7]. A study of 988 participants investigating the risk factor of exposure to blood and body fluids among laboratory workers found that 64% of healthcare workers had been exposed to blood and body fluids at least once during their career. Needle recapping was identified as the main factor contributing to sharps injuries. It was also found that 28% of injured healthcare workers did not use personal protective equipment, and 67% did not seek medical attention after their injury [8].

GDDCs are classified as Biosafety Level 3 (BSL‐3) laboratories according to the World Health Organization (WHO) risk groups of BSL‐1, BSL‐2, BSL‐3, and BSL‐4 [9]. Biosafety levels are designed to protect both the environment and laboratory personnel during laboratory activities. These levels also set standards for the precautions that must be taken to minimize the risks present [10].

The primary motivation for this study stems from the inadequacy of classifying GDDCs solely on the basis of their biosafety levels from an occupational health and safety (OHS) perspective. In classifying laboratories according to biosafety levels, the primary focus has been on the biological threats posed by the infectious agents handled in the laboratory. However, ensuring laboratory safety requires the effective integration of processes such as “physical safety”, “chemical safety”, “biosafety”, “waste management”, “personal protective equipment”, and “risk management”. This need for a multifaceted approach highlights the limitations of relying solely on biosafety level classification [11].

This study aims to systematically analyze OHS practices in GDDCs in Istanbul, with a focus on identifying the hazard groups encountered in these centers and examining their implications for risk management. The study will identify areas where safety protocols are inadequate and develop strategic recommendations to improve safe working conditions in these centers. In addition, the findings will serve as a reference for similar health laboratories and contribute to the improvement of general OHS standards.

## 2 Materials and Methods

### 2.1 Location and Population of the Study

This study was conducted in GDDCs in Istanbul. The city of Istanbul was selected as the study area because of the high number of laboratories and its status as a metropolis. According to the list of GDDCs obtained from the Istanbul Provincial Health Directorate, there are a total of 30 laboratories, including six public hospital laboratories and 24 private laboratories. Preliminary discussions for the laboratory visits revealed that four laboratories were not actively operating and another four did not respond positively or negatively to the visit request. After excluding laboratories that were not performing diagnostic tests or did not respond to the visit request, physical visits were conducted at the remaining 22 laboratories. Of these, seven laboratories refused to participate in the study, and the study was finally carried out in 15 laboratories with a total of 75 staff.

### 2.2 Research Data

**FIGURE 1:**
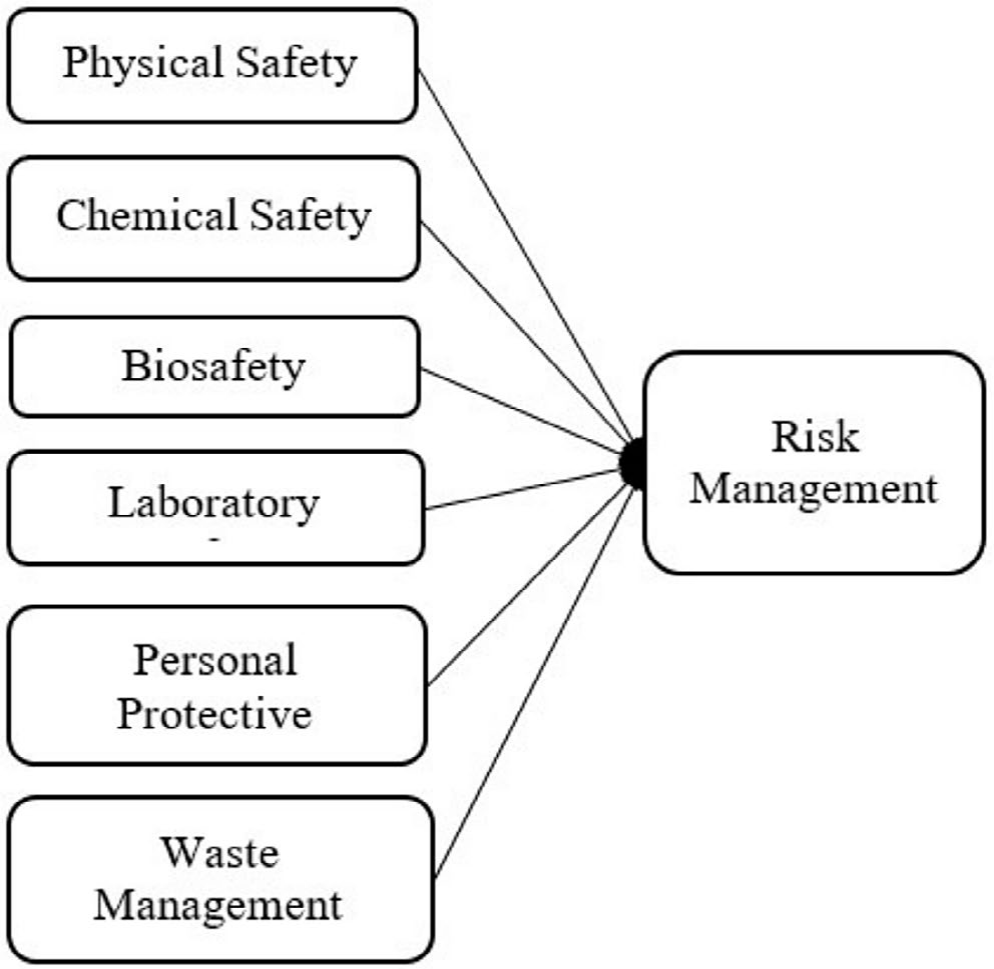
Research model.

Research data was collected using a research data form. The study included a total of 49 statements in seven categories: “physical safety” with four statements, “chemical safety” with 12 statements, “biosafety” with eight statements, “laboratory safety” with four statements, “personal protective equipment” with seven statements, “waste management” with five statements, and “risk management” with nine statements. Participants responded using a 7‐point Likert scale. Before the scale was used, an informed consent form was signed by all 75 laboratory staff who participating in the study. Accordingly, the research model is shown in Figure 1.

### 2.3 Statistical Analysis

A data form was used as the data collection method, and after fieldwork, the collected data was transferred to SPSS (v.25) where regression and correlation analyses were carried out. Analyses were conducted at the 95% confidence level.

Regression analysis was used to determine the interactions between the dimensions. The risk management dimension was selected as the dependent variable and its levels of influence with other dimensions were examined. The impact of physical safety, chemical safety, biosafety, laboratory safety, personal protective equipment and waste management on risk management was 34.8, 54.4, 51.8, 35.1, 56.3, and 54.7 respectively. The direct and positive effect of all dimensions on risk management confirmed the hypothesis of the study that “Hazard dimensions have a direct and positive effect on risk management”.

Correlation analysis was also used to determine the relationships between the dimensions. When examining the relationships between the dimensions, it was found that 18 of the 21 relationships identified were “very strong”, while 3 were “strong”. When ranked by strength, the relationship between “laboratory safety” and “physical security” was the strongest, while the relationship between “physical security” and “risk management” was the weakest.

In this study, Principal Component Analysis (PCA) was used to understand the variability in the data set and to summarize the relationships within the data [12]. PCA reduced the 6‐dimensional data to three components. Finally, the k‐NN algorithm was applied to demonstrate the accuracy of the PCA‐based data arrangement in classifying genetic disease diagnostic center laboratories [13].

## 3 Results

### 3.1 Dimensionality Reduction of the Dataset

In this study, PCA was used to understand the variability within the dataset and to summarize the relationships within the data. PCA analysis aims to reduce the complexity of data analysis and reveal underlying structures by summarizing the multidimensional data in the dataset with a smaller number of components [14].

The component matrix obtained by PCA is shown in Table 1. This method summarizes complex structures within the dataset and deepens our understanding of data analysis. The PCA component matrix strengthens the analytical framework of this study by revealing significant relationships and structures within the dataset.

**TABLE 1:** Component matrix.

	Components/factors		
	1	2	3
Personal protective equipment	0.943		
Waste management	0.894		
Physical safety	0.869		
Chemical safety		0.729	
Laboratory safety		0.684	
Biosafety			0.803

The first component shows a positive relationship between variables such as “personal protective equipment”, “waste management”, and “physical security”. This component may represent a cluster based on factors such as personal protective equipment, waste management, and physical security.

The second component shows a positive relationship between variables such as “chemical safety”, “laboratory safety”, and “biosafety”. This component may represent a different cluster that includes laboratory safety issues related to chemical safety. The relationships between these variables, particularly those related to safety concerns, support the core analytical findings of the study.

The third component, identified as “biosafety”, appears to represent a separate cluster. Given that GDDCs are currently classified according to their level of biosafety, the emergence of biosafety as a single category indicates that this study is consistent with existing laboratory classification systems.

A variance explanation table is used to show the contribution of each PCA component to the total variance and the cumulative total variance [15]. According to the results of the PCA analysis, the explanation of the total variance is shown in Table 2.

**TABLE 2:** Total variance explained.

Components/factors	Initial Eigenvalues			Sum of squared loadings		
	Total	% of variance	Cumulative %	Total	% of variance	Cumulative %
1	2.590	43.166	1	2.590	43.166	1
2	1.244	20.726	2	1.244	20.726	2
3	1.080	18.007	3	1.080	18.007	3
4	0.672	11.198	4	0.672	11.198	4
5	0.268	4.475	5	0.268	4.475	5
6	0.146	2.428	6	0.146	2.428	6

As can be seen in Table 2, the first component explains 43.166% of the variance, giving a cumulative variance of 43.166%. The second component explains 20.726% of the variance, giving a cumulative variance of 63.892%. These first two components account for a significant proportion of the variability within the dataset and outline the structural patterns within the data. The third component explains 18.007% of the variance, giving a cumulative variance of 81.899%. These results indicate that the majority of the variability in the dataset is represented by the first three components.

### 3.2 Clustering of Genetic Disease Diagnostic Centers

The k‐NN algorithm is a machine learning method that determines the class or value of a data point by considering the classes or values of its nearest neighbours. It can be applied to both classification and regression problems. The core principle of the algorithm is that similar data points typically have similar classes or values [16]. k‐NN differs from non‐representative methods in that it incorporates all data directly into the learning process and uses the entire data set during the prediction phase [17].

The data set, reduced to three dimensions by PCA, was subjected to a clustering analysis process using the k‐NN algorithm. The value of “k” was obtained by PCA and set to three in the analysis algorithm. In this model, the number of neighbours was set to three. The number of neighbours represents the number of nearest neighbours that the model will consider when classifying a data point. Lower numbers of neighbours can make the model more sensitive and potentially overfit, while higher numbers of neighbours can lead to a more generalized model, which can sometimes result in underfitting [18].

In addition, this model used Euclidean distance as the distance metric. Euclidean distance calculates the linear distance between two points and is one of the most commonly used distance metrics. This metric is particularly effective when the data are continuous and on the same scale [19].

The distance weighting of the model was also set to “equal”, meaning that all neighbours were considered with equal weight. In some applications, closer neighbours can be given higher weights to increase prediction accuracy [20]. However, in this model, equal weighting of all neighbours was preferred.

In this model, all six individual features were selected. Feature selection is a critical step in improving model performance and avoiding overfitting. Careful selection of the features used has a positive effect on the overall performance and accuracy of the model [21].

The analysis showed that the k‐NN algorithm achieved a validation accuracy of 93.33% in reducing the six‐dimensional research dataset to three dimensions. In the k‐NN algorithm, “accuracy” is a metric that evaluates the classification performance of the model and represents the ratio of correctly classified samples to the total number of samples. Accuracy indicates how accurate the model's predictions are and is typically expressed as a percentage. The higher the accuracy of a classification model, the better the performance of the model is considered to be [22]. The accuracy of the model was determined to be 93.3%, indicating high performance. Careful selection of hyperparameters and data standardization positively influenced the performance of the model. Future studies could evaluate the overall effectiveness of the model more comprehensively by considering additional performance metrics.

## 4 Discussion

Classification studies using PCA and k‐NN algorithms have successfully reduced the dimensionality of the dataset and improved classification accuracy. The use of these methods is also widely acknowledged in the existing literature. Previous studies have shown that PCA and k‐NN algorithms can be effectively used for data analysis and classification tasks [23]. Similarly, the effectiveness of PCA in simplifying data sets and minimizing information loss has been highlighted [24].

Based on the results of this study, GDDCs were classified into three groups according to their level of OHS: Level 1, Level 2, and Level 3. Level 1 laboratories were clustered together based on similarities in personal protective equipment, waste management, and physical security dimensions. Level 2 laboratories formed a separate cluster due to their common characteristics in the chemical safety and laboratory safety dimensions. Laboratories representing the newly defined Level 3 category were clustered based on their proximity in the biosafety dimension.

Public health laboratories, including GDDCs, are classified into four groups (BSL‐1, BSL‐2, BSL‐3, and BSL‐4) based on their biosafety levels in the existing literature [25]. This classification is based solely on the risk group of the biological agent and is the only classification available to laboratories. The analyses performed in this study showed that the genetic disease diagnostic laboratories involved in the research clustered into a single group (Level 3) based on their biosafety characteristics. This finding is consistent with results reported in the existing literature.

A systematic review of PubMed articles published between 2006 and 2021 focusing on BSL‐3 laboratories, including GDDCs, found that despite precautions against biological agents, laboratory accidents still occur due to exposure to various risk factors [26]. In addition, another study of BSL‐1, BSL‐2, and BSL‐3 laboratories found that many laboratories did not meet the standards of the biosafety level they were supposed to represent [27]. These findings support the core premise of this study: the classification of laboratories based on biosafety levels alone is not sufficient to determine occupational safety levels. The results of previous studies support the core premise of this study: that the classification of laboratories based on their biosafety level alone is insufficient to determine their occupational safety level. In addition, it was found that two other factors, in addition to the biosafety dimension, significantly influence the occupational safety and health levels of laboratories.

Laboratories classified as Level 2 in this study were grouped in this cluster because of their similar representation in the chemical safety and laboratory safety dimensions. A new risk management strategy was developed in a study that examined the relationship between chemical safety and laboratory safety. The results of the study, conducted in chemical laboratories, showed that chemical safety and laboratory safety shared 64.9% of common hazards and risks [28]. Similarly, another study in this area examined the incidence of laboratory accidents and the factors contributing to them and concluded that chemical safety was the most important factor leading to accidents [29]. These findings are consistent with the results of this study, where chemical safety and laboratory safety were grouped together as a separate dimension and classified as Level 2 laboratories.

The extensive use of chemicals in the diagnostic processes performed in GDDCs supports the grouping of laboratory safety and chemical safety into a single dimension. In addition, studies indicating that a significant proportion of workplace accidents and occupational diseases in GDDC laboratories are caused by chemical hazards [30] are consistent with the finding that chemical safety and laboratory safety are grouped together as Level 2.

For laboratories grouped as Level 1, a literature review was conducted to examine studies highlighting the relationships between personal protective equipment (PPE), waste management, and physical safety. One study reported that between 2016 and 2020, the global PPE market will experience a compound annual growth rate of 6.5%, increasing from $40 billion to $58 billion. It concluded that the increase in PPE production and distribution, particularly in countries with underdeveloped infrastructure, has led to a corresponding increase in waste streams, coupled with health and environmental risks throughout the waste management chain [31]. Another study focusing on the use of masks and gloves during the COVID‐19 pandemic found that PPE had become a significant waste category. The study highlighted the need for more information on how PPE should be procured and disposed of. It was suggested that the use of color‐coded waste collection bags for different types of PPE and the implementation of automated PPE waste collection systems could significantly improve waste management practices [32]. These findings highlight the strong link between the use and disposal of PPE and waste management, and emphasize the importance of integrating waste management considerations into PPE‐related processes.

### 4.1 Conclusions

The analyses conducted in this study have provided a detailed overview of the current status of OHS protocols in GDDCs, the risks faced by workers, and the areas within these protocols that need improvement. The findings provide comprehensive recommendations for improving OHS standards and help identify strategic steps to prevent laboratory accidents. Finally, the study classified GDDCs into three groups based on their OHS levels: Level 1 laboratories, Level 2 laboratories, and Level 3 laboratories.

## Conflicts of Interest

The authors declare no conflicts of interest.

## Data Availability

The data supporting the findings of this study are not publicly available due to privacy and ethical restrictions. In accordance with the informed consent obtained from the participants, their institutional affiliations and personal findings are confidential and will not be shared. The data can be made available upon reasonable request to the corresponding author, provided the request complies with ethical guidelines and privacy policies.
