# Correction to “Investigation of Occupational Health and Safety Levels in Genetic Disease Centers in Istanbul”

**DOI:** 10.1002/jcla.70166

**Published:** 2026-01-14

Caner V. Tanir F. “Investigation of Occupational Health and Safety Levels in Genetic Disease Centers in Istanbul,” *Journal of Clinical Laboratory Analysis* (2025) 39: e70015.

The Acknowledgement section was inadvertently omitted from the published article. To comply with academic regulations and ensure transparency, the Acknowledgement section below has been added to the proof.

Acknowledgements

“This study was produced from the PhD thesis of Vedat Caner, titled ‘Investigation of Occupational Health and Safety Levels in Genetic Disease Centers in Istanbul’ (Turkish: İstanbul'daki Genetik Hastalık Tanı Merkezlerinin İş Sağlığı ve Güvenliği Düzeylerinin Araştırılması), completed at Cukurova University in 2024, under the supervision of Prof. Dr. Ferdi Tanır.”

We apologize for this error.

